# Dual Differentiation-Exogenous Mesenchymal Stem Cell Therapy for Traumatic Spinal Cord Injury Repair in a Murine Hemisection Model

**DOI:** 10.1155/2013/928982

**Published:** 2013-08-20

**Authors:** Hai Liu, Edward M. Schwarz, Chao Xie

**Affiliations:** ^1^Department of Orthopaedics and Rehabilitation, Center for Musculoskeletal Research, University of Rochester Medical Center, 601 Elmwood Avenue Box 655, Rochester, NY 14642, USA; ^2^Department of Neurosurgery, Beijing Tiantan Hospital Affiliated Capital Medical University, 6 Tiantan Xili, Dongcheng District, Beijing 100050, China; ^3^Joint Center for Musculoskeletal Research of Zunyi Medical University & University of Rochester Medical Center, Zunyi Medical University, 201 Dalian Road, Zunyi, Guizhou 563003, China

## Abstract

Mesenchymal stem cell (MSC) transplantation has shown tremendous promise as a therapy for repair of various tissues of the musculoskeletal, vascular, and central nervous systems. Based on this success, recent research in this field has focused on complex tissue damage, such as that which occurs from traumatic spinal cord injury (TSCI). As the critical event for successful exogenous, MSC therapy is their migration to the injury site, which allows for their anti-inflammatory and morphogenic effects on fracture healing, neuronal regeneration, and functional recover. Thus, there is a need for a cost-effective in vivo model that can faithfully recapitulate the salient features of the injury, therapy, and recovery. To address this, we review the recent advances in exogenous MSC therapy for TSCI and traumatic vertebral fracture repair and the existing challenges regarding their translational applications. We also describe a novel murine model designed to take advantage of multidisciplinary collaborations between musculoskeletal and neuroscience researchers, which is needed to establish an efficacious MSC therapy for TSCI.

## 1. Introduction

With almost 12,000 new spinal cord injuries (SCI) occurring every year in the United States alone, near half a million chronic SCI patients suffer the long term consequences of this devastating injury. Since the major disabilities from SCI are neurological deficits, neural regeneration remains the priority. Consequently, other aspects of SCI, such as vertebral fracture reconstruction, receive less attention. Thus, one major limitation in this field that has contributed to the lack of progress has been the absence of multidisciplinary cooperation between neuroscientists working towards nerve regeneration and orthopaedic investigators working with mesenchymal stem cells (MSCs) for bone repair [1].

One of the most challenging aspects of treating injuries to the spinal cord is the multitude of problems that need to be addressed to restore normal function. These include neural cell death, limited axon regeneration, inflammation and scar formation, and disruption of the neurovascular supply and loss of structural support from the surrounding vertebra. Thus, any therapeutic approach aimed at SCI tissue regeneration requires a coordinated approach in which neural repair is accompanied by fracture repair and revascularization of newly formed tissues [2].

Several types of cell transplants have been proposed for SCI and fracture repair, including stem cells and their differentiated progeny, with the purpose of directly replacing lost neurons, oligodendrocytes, and osteoblasts, respectively. MSCs have shown great potential to enhance osteogenesis and chondrogenesis for spinal fusion repair. Furthermore, transplanted MSCs have the ability to differentiate into osteoblasts in the presence of specific bioactive factors, such as stromal cell-derived factor-1/CXCR4, nutrients, and extracellular matrix in the MSC/hydroxyapatite/type I collagen hybrid graft [1, 3–5]. However, controversy in the field remains over the extent of exogenous MSC contribution to neuronal regeneration, despite evidence from animal models and human specimens data showing the potential of neuronal differentiation [6–12]. Thus, the development of a cost-effective animal model to definitively answer this question is warranted.

## 2. TSCI Murine Models for Cell-Based Therapy

The fundamental events of SCI can be divided into four main stages: the immediate, acute, intermediate, and chronic phases [13]. To fulfill its final neurological outcomes, a reproducible TSCI model is essential that can be either improved or deteriorated by the intervention of interest [14, 15]. For small animals, such as mice and cats, the most widely accepted models include epidural balloon compression [14, 16], weight-drop contusion injury [17, 18] and modified aneurysm clip crash [19, 20], and hemisection removal critical defect and hemicontusion force [21]. 

### 2.1. Hemisection Model of Unilateral Injury

**Figure 1:**
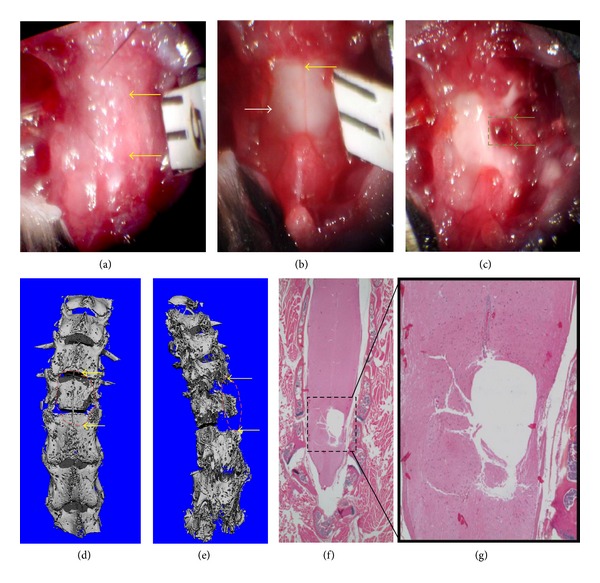
A murine laminectomy and hemisection model of TSCI. Development of a murine laminectomy and hemisection model of TSCI was achieved using protocols approved by the University of Rochester Committee for Animal Resources (IACUC). After the animal is anesthetized, a laminectomy is performed to remove thorax 11 lamina (a), then the dura is opened to expose the spinal cord (b), and, finally, a hemisection lesion is performed to generate a 2 mm defect in the right half side of the spinal cord (c). Postoperatire dorsal view (d) and lateral view (e) of micro-CT scans of the spine; 5x (f) and 20x (g) micrographs of H&E stained histology sections are presented to illustrate the vertebral bone and spinal cord defects that generated in this model, respectively.

Although hemisection of the spinal cord is not a clinical relevant model, our interests in this field are focused on understanding the effects of transplanted MSCs on simultaneous angiogenesis, osteogenesis, neuronal survival, axonal growth, and remyelination following TSCI. Thus, in addition to being a highly reproducible injury and response to host response to TSCI, the hemisection model provides clear injury section boundary for radiological and histological outcomes to assess transplanted MSCs proliferation and neuronal differentiation. To this end, we have developed a novel hemisection-unilateral TSCI model in mice (Figure 1). The major advantage of this model is that it allows researchers to transfer synthetic biomaterials with or without exogenous MSCs locally to overcome secondary damage to the SCI. These transferred MSCs are known to mediate healing by orchestrating a favorable environment for parenchymal cell survival and stimulating cell bridges within the traumatic centromedullary cavity. Following a laminectomy, the surgical procedure involves longitudinal exposure of the dura mater, and then a spinal cord hemisection is made at the appropriate spinal cord level, which is then followed by the removal of 2-3 mm hemicord segment along the midline using microscissors. After cell transplantation, the dura, muscle, and fascia are sutured separately using methods that have been previously described [22, 23].

### 2.2. Modified Aneurysm Clip Crash

Compared to other TSCI murine models, modified aneurysm clip could mimic an initial impact plus persisting compression. With a gradient clinical relevant compression that reminds the sparing of white matter tracts, this model can provide information about surviving tracts and residual motor function. However, it suffers from an ~10% mortality rate during the injury procedure, especially during laminectomy, due to excessive blood loss and incidence of anesthetic sensitivities. A longitudinal incision is made on the midline of the back to expose the superficial muscle layers and then bluntly dissect vertebrae attached muscle. A laminectomy is performed on the target vertebrae and part of pedicles with a pair of microscissors. An extradural path between the spinal cord and the vertebral body is created to pass the lower blade of modified aneurysm clip underneath the spinal cord and hook on its upper blade to make a ventral and dorsal compression [19, 20, 24].

### 2.3. Weight-Drop Contusion Technique

Fifty percent of human spinal cord injuries contain some white matter tissue that is spared, which contains uninjured axonal projections. Friedenstein and colleagues investigated the electrophysiological and morphological data from 85 patients and 27 adult rats that indicated the weight-drop contusion model in rat and demonstrated that this model can serve as an adequate animal model for the effects of new treatment strategies TSCI [25]. To produce the model, T10 laminectomies were performed. While the vertebral column was stabilized by Adson forceps, the impactor probe was positioned 2–4 mm above the spinal cord. An impact force of 150 kilodyne was delivered to the exposed spinal cord through the intact dura with an Infinite Horizons impactor to create a moderate severity contusion injury [26, 27]. 

### 2.4. Epidural Balloon Compression Injury

To produce precise quantities submaximal damage SCI, Vanický and colleagues modified a saline-filled Fogarty catheter subdural compression to epidural compression and could customized the gradient of injury [14, 28]. The brief procedures include a 2-French Fogarty arterial embolectomy catheter (Baxter Healthcare Corporation, Irvine, CA) that was inserted in the epidural space at the T10 level and moved rostrally for 2 metameric levels before being inflated with a 15 mL distilled water volume and left in place for 5 minutes. The balloon was then deflated and carefully removed. Skin and muscle were carefully closed in two layers. Histological cross-section of spinal cord has shown a correlated damage of white and gray matter significantly with gradient compression. 

## 3. Current Advances in MSC-Based Therapies for TSCI and Fracture Repair and the Frontier of MSC Dual Differentiation

Since MSCs were first isolated by Friedenstein and colleagues in 1968 [25], the plastic-adherent bone marrow derived MSCs are typically characterized by their cell surface markers positive for Stro-1, CD29, CD73, CD90, CD105, CD166, and CD44 and negative for CD34, CD45, CD14, CD11b, CD19, CD79a, and HLA-DR [29]. The fate of these MSCs is known to be limited in serial passages due to the lack of alternative lengthening of telomeres (ALT), which results in telomeric DNA shortening at each cell division and eventually senescence [30–32]. However, prior to its 10th passage, exogenous MSCs retain their stemness and proliferative capacity to facilitate bone repair such as fracture nonunion, osteogenesis imperfecta and hypophosphatasia [29, 33–38]. 

Another important property of MSCs is that they can terminally differentiate into multiple lineages including osteoblasts, chondrocytes and myoblasts, fibroblasts, adipocytes, and oligodendrocytes [39–46]. We and others have shown definitive MSC-mediated osteogenesis in murine models of fracture and structural allograft healing. Rashidi et al. compared MSCs with three nonosteogenic cell lines of HEK293, HeLa, and NTera and found that MSCs are uniquely capable of depositing mineral through an independent mechanism of established dexamethasone or bone morphogenetic protein signaling [47]. 

In contrast, experimental evidence formally demonstrating MSC neuronal differentiation remains controversial, in part because MSCs are derived from the mesoderm, while neurons are derived from the ectoderm. However, in support of the MSC-neuron differentiation theory, there are numerous publications showing that neuronal marker expression in MSCs can be induced following stimulation with epidermal growth factor (EGF) and basic fibroblast growth factor (bFGF) [48–51]. Deng and collogues even reported that MSCs significantly increase expression of the astrocyte-specific glial fibrillary acidic protein spontaneously in the absent of cytoplasmic cyclic AMP, which is a neuronal specialized induction reagent [51]. 

Collectively, this evidence indicates that MSCs have dual differentiation capability. For clinical transplantation, the ideal administration mode of MSC transplantation is intravenous or intraoperative administration of an MSC pre-seeded biomaterial scaffolds. Clinical studies evaluating the efficacy of exogenous MSC therapy for bone repair have shown significant improvement of bone mineral density and linear bone growth in patients [34–38]. In contrast, the efficacy of MSC-mediated neuronal recovery remains to be formally evaluated by functional assessments and histological confirmation. Thus, experiments in the murine model described here should be able to answer these important questions in the future.
